# CircNDC80 promotes glioblastoma multiforme tumorigenesis via the miR-139-5p/ECE1 pathway

**DOI:** 10.1186/s12967-022-03852-3

**Published:** 2023-01-12

**Authors:** Yuhang Wang, Binbin Wang, Fengqi Zhou, Kun Lv, Xiupeng Xu, Wenping Cao

**Affiliations:** Department of Neurosurgery, The First Affiliated Hospital of Nanjing Medical University, Nanjing, 210000 Jiangsu China

**Keywords:** CircNDC80, Glioblastoma, miR-139-5p, ECE1, GSCs

## Abstract

**Background:**

Circular RNAs (circRNAs) have been shown to be essential for the emergence and growth of different cancers. However, further research is required to validate the function of circRNA in glioblastoma (GBM).

**Methods:**

CircNDC80 expression in both normal brain tissues (NBTs) and glioma tissues was determined using real-time PCR. The impact of circNDC80 on GBM cell proliferation, migration, and invasion was then confirmed by CCK-8, colony formation, EdU incorporation, Transwell, and wound healing assays. To determine how circNDC80 affects the capacity of glioma stem cells (GSCs) to maintain their stemness and self-renewal, a CellTiter-Glo assay, clonogenic assay and extreme limiting dilution assay were utilized. To ascertain the impact of circNDC80 in vivo, intracranial xenograft models were established.

**Results:**

When compared to NBT, glioblastoma tissue had a higher level of circNDC80 expression. In functional assays, circNDC80 promoted glioblastoma cell proliferation, migration, and invasion, while sustaining the stemness and fostering the self-renewal of glioma stem cells. In addition, a dual luciferase reporter assay and circRIP were used to verify that circNDC80 simultaneously affects the expression of ECE1 mRNA by sponging miR-139-5p, and a rescue experiment was used to verify the above results further.

**Conclusions:**

According to our research, circNDC80 is an oncogenic factor that promotes glioblastoma through the miR-139-5p/ECE1 pathway. This implies that circNDC80 may be employed as a novel therapeutic target and a possible predictive biomarker.

**Supplementary Information:**

The online version contains supplementary material available at 10.1186/s12967-022-03852-3.

## Introduction

The most common primary brain tumour is glioma [1]. Glioblastoma (GBM), the most severe malignancy of the central nervous system, is the most prevalent brain tumour [2]. The standard treatment is surgery and postoperative radiation [3–5]. This cancer is highly invasive, metastasizes widely, and mixes with healthy brain tissue, making surgical removal of the tumour impossible [5], and less than 3% of glioblastoma patients are alive 5 years after diagnosis [6, 7].

Previous studies have shown that tumour growth is driven by a small number of cancer stem cells hidden in the tumour [8]. This finding also explains clinical observations such as the almost inevitable recurrence and metastasis of tumours after initial successful chemotherapy or radiotherapy [9, 10]. Current studies on the role of cancer stem cells have been conducted in cancers such as leukaemia, breast cancer, and colorectal cancer, but little is known about glioma stem cells (GSCs) [11–14].

A specific kind of noncoding RNA molecule is the circular RNA (circRNA) [15]. CircRNA molecules have a closed circular form that is unaffected by RNA exonucleases, in contrast to typical linear RNA, which has a 5′ and a 3′ end [16, 17]. CircRNAs have been shown to have a variety of roles, including roles as transcriptional regulators, RNA-binding proteins (RBPs), and microRNA (miRNA) –sponges [18]. Recent research has shown that circRNAs are linked to many different cancer types by sponging miRNAs and suppressing miRNA functions in the breast [19, 20], liver [21], and lung [22]. The competitive endogenous RNA (ceRNA) mechanism is the term describing this mode of action [23]. Numerous miRNAs have been identified to have roles in the development and spread of glioma [24, 25].

Studies have shown that a series of thiol ECE1 (endothelin converting enzyme 1) inhibitors exert rapid inhibitory effects on human glioblastoma cells [26]. This effect is not related to insufficient extracellular ET-1 production or reduced binding of ET-1 to its membrane receptor(s) [27]. However, it may be influenced by other ECE1 substrates or by the intracellular functions of ET-1 and/or ECE1 in regulating cell proliferation. Four isoforms of ECE1 with different cytoplasmic N-termini are expressed [28]. The ECE1c isoform was recently discovered to be involved in cancer cell proliferation and invasiveness in a colorectal cancer cell invasiveness study. The protein kinase CK2, also called casein kinase 2, phosphorylates the N-terminus of ECE1c, increasing its stability [29]. According to previous studies, cancer stem cell (CSC) properties, such as increased stem cell gene expression, chemotherapy resistance, self-renewal, colony formation, and sphere formation, as well as enhanced tumour growth and metastasis in vivo, are exhibited by colorectal cancer cells with upregulation of ECE1 [30].

Our recent study revealed that circNDC80 expression was elevated in glioma tissues and cell lines and related to the prognosis of glioma. In addition, the role and mechanism of circNDC80 in gliomas were studied. Functionally, downregulation of circNDC80 led to decreases in GBM cell proliferation, migration, and invasion and GSC maintenance. CircNDC80 may act as a molecular sponge for miR-139-5p, upregulating the ECE1 oncogene to hasten the development of GBM. As a result, circNDC80 may be used as a new therapeutic target and prognostic biomarker for glioma.

## Materials and methods

### Clinical specimens

At the First Affiliated Hospital of Nanjing Medical University, glioma patient specimens (n = 45) were collected by surgical resection. Normal brain tissue samples (NBT) from severe traumatic brain injury patients (n = 8) undergoing craniotomy decompression and cerebrovascular malformations were also collected as negative controls. After surgical excision, samples were promptly frozen in liquid nitrogen and preserved there. Experienced pathologists identified glioma samples using World Health Organization (WHO) diagnostic standards. Patients and their families granted informed consent for all tissue collection, and the ethics committee of Nanjing Medical University authorized our study technique. Specific patient information is provided in Additional file 5: Table S2.

### Cell lines and cell culture

Glioma cell lines U251, LN229, U118, U87, and T98G were bought from the Cell Bank of the Chinese Academy of Sciences (Shanghai, China). In a DMEM media with 10% FBS, the primary GBM cell line (pGBM-1) was maintained after being generated from surgical specimens of GBM. The American Type Culture Collection (ATCC) provided normal human astrocytes (NHAs). In the laboratory of the common platform of the Wutai Campus of Nanjing Medical University, all cell lines were kept in liquid nitrogen containers.

### Transfection

GenePharma (Shanghai, China) created mimics of siRNAs and miRNAs. Following is a list of the oligonucleotide sequences.:

#sh-RNA-1: 5′-CAGTAAAGTGTATTGAATGCA-3′;

#sh-RNA-2: 5′-AGTAAAGTGTATTGAATGCAA -3′;

#sh-RNA-3: 5′-AAGTGTATTGAATGCAATGAT-3′.

The aforementioned sequence was cloned into the RioBio pcDNA3.1 vector (Shanghai, China) to make pcDNA-cNDC80. This allowed for the regulation of the expression level of cNDC80. GenePharma provided the miR-139-5p inhibitor (anti-miR-139-5p), miR-139-5p mimic (miR-139-5p), and matching controls (anti-NC and miR-139-5p-NC) (Shanghai, China). GenePharma supplied lentiviral vectors with anti-miR-139-5p or anti-NC and shRNA or shCONT (Shanghai, China).

### Quantitative RT–PCR and RNase R treatment

TRIzol (Invitrogen) reagent was utilized to harvest total RNA from tissues and cells. Following the manufacturer's instructions, PrimeScript RT Master Mix (TaKaPa) was applied to reverse-transcribe circRNA, miRNA, and mRNA. Using the 2^–ΔΔCT^ technique and 18S rRNA as the internal reference, the relative quantification of circNDC80, mNDC80, miR-139-5p, and ECE1 were calculated. CircNDC80, mNDC80, ECE1, and miR-139-5p were standardized to GAPDH and U6, respectively. Three separate runs of these responses were made. On a StepOnePlus Real-Time PCR System (Thermo Fisher, USA), quantitative PCR tests were run, and the results were assessed on AGE (agarose gel electrophoresis). The Additional file contains a list of all primer sequences (Additional file 4: Table S1).

The RNeasy MinElute Cleaning Kit (Qiagen, Dusseldorf, Germany) was used to purify RNA after it had been subjected to 3 U/mg of RNase R. After that, qRT–PCR was employed to ascertain the level of mNDC80 and cNDC80 expression.

### *RNA fluorescence *in situ* hybridization (FISH)*

RiboBio (Guangzhou, China) produced the miR-139-5p FAM-labeled and cy3-labeled probes for cNDC80. Following 4% paraformaldehyde fixation for twenty minutes at room temperature and PBS washing, cell lines were grown in 10% fetal bovine serum. After that, the membranes were dissolved in 0.5% triton-X-100 PBS for 5 min at room temperature. Prehybridization buffer was immediately applied to the cells, and then the probe was added to the hybridization buffer overnight. Following that, cells were washed three times with saline-sodium citrate (SSC) at 42 °C for 5 min. After staining the nuclei with DAPI for ten minutes, the signal from the probes was examined using a fluorescence in situ hybridization kit (RiboBio, Guangzhou, China).

### CCK-8 assay

The proliferation of U87 and pGBM-1 was discovered using the CCK-8 kit (RiboBio, China). A 96-well plate with 100 μl of media each well and six duplicate wells included was filled with 1000 cells. A culture system was created by mixing 10 μl of CCK-8 reagent with 90 μl of DMEM media. Then, each well received 100 μl of this system, and it was cultured for 1.5 h. This experiment was carried out over 1, 2, 3, and 4 days.

### Colony formation assay

Before being frozen in 4% paraformaldehyde for a further 10 min, cells (3 × 10^3^) were cultured for 14 days at 37° C in an incubator with 5% CO_2_ before being seeded on cell culture plates (35 mm, Corning, USA). The number of clones was recorded on the clear film on the grid after staining with crystal violet dye (Beyotime, China) for 20 min.

### 5-Ethynyl-20-deoxyuridine (EdU) assay

Cells (2 × 10^4^) were seeded onto 96-well plates as previously reported and cultured overnight at 37° C in an incubator with 5% carbon dioxide for the EdU experiments [31]. Cells were fixed using 4% paraformaldehyde fixator, the Apollo dye solution (RiboBio) and the nuclei were stained with DAPI (Invitrogen). Images were then captured using a fluorescence microscope (Olympus) after culturing for 2 h in EdU (RiboBio, Guangzhou, China).

### Transwell assay

As reported earlier, glioma cell invasion and migration were measured using Transwell [31, 32]. The main change was the adoption of a modified 8-m pore size culture method and matrigel coating for the top chamber. Serum-free media and 10%FBS medium were layered at the bottom of Transwell Inserts before transfected cells were planted inside. Cells were stained with crystal violet after being in development for 24 h before being fixed with 4% paraformaldehyde solution. The number of cells infiltrating the holes was determined using ImageJ software after pictures taken under an Olympus microscope were obtained. Three times each were done for every experiment.

### Wound-healing assay

In order to compare the effects of transfection on GBM cell lines, several were seeded in 6-well plates and grown to confluence. Utilizing a 200 µL pipette tip, cell monolayers were scraped. At 0 h, 24 h, and 48 h, respectively, cell movement was recorded using an Olympus microscope (Olympus, Tokyo, Japan).

### Western blot assay

Different sets of GBM cells were lysed with RIPA buffer (KeyGEN, Jiangsu, China) below the ice. The protein lysates were then centrifuged at 12,000 RPM, and the supernatant was collected and electrophoresed on a 10%SDS polyacrylamide gel, which was subsequently transferred to a PVDF membrane (Millipore, USA) and blocked with 5% skim milk. Subsequently, primary antibodies were added at 4℃: anti-ECE1 (abcam, #ab71829,1:1000), anti-Sox2 (#ab97959); Cell Signaling Technology: anti-N-cadherin (# 13116,1: 1000), anti-Vimentin (# 5741, 1:1000), anti-CyclinD1 (# 55506, 1:1000), anti-Oct4 (# 2750, 1:1000), anti-Nanog (#4903, 1:1000), anti-GAPDH (#5174,1:1000) was used overnight. Subsequently, anti-rabbit IgG secondary antibody (Cell Signaling Technology, #7074, 1:1000) was applied to the membranes for 2 h at room temperature. Finally, the gel imaging system (Bio-Rad, USA) was used for exposure.

### Glioma stem cell viability assay

The pGBM-1-GSCs and U87-GSCs that were in excellent condition were gathered, digested, resuspended, spread into large dishes, and incubated for 48 h at 37 ℃ in a 5% CO_2_ incubator. GSCs cells were harvested and organized into a centrifuge tube at 600 rpm for 5 min to remove dead cells. After discarding the supernatant, 2 ml of trypsin was added and resuspended for 5 min. Subsequently, the supernatant was removed by centrifugation again, and 2–3 ml of the medium was added and resuspended. Cells were counted (1 × 10^3^ cells per well), and 1000 cell volumes were calculated. Then 200 ul of medium and the corresponding 1000 cell volumes were added to each well, and the control and experimental groups were repeated for 6 wells each. Finally, 30 ul of the reagent (CellTiter-Glo, Promega, USA) was added, shaken for 5 min with a shaker, and placed in a microplate reader (Thermo Scientific, USA) for activity detection.

### GSCs sphere formation assay and extreme limiting dilution assay

The cells in good condition (U87-GSCs, pGBM-1-GSCs) were digested and collected by centrifugation to remove the serum-containing medium. They were then washed twice with PBS. Using an Olympus microscope (Olympus, USA), sphere formation was examined for 10 days while cells were cultivated in ultra-low adhesion cell culture plates (about 1 × 10^3^ cells per well in 6-well plates). The sphere formation rate was then estimated. For extreme limiting dilution assay, GSCs with the specified alterations or treatments were divided into individual cells and plated in 96-well plates at densities of 5, 10, 20, 40, or 100 cells per well for limit dilution tests. Each well's tumor sphere development was evaluated after seven days of incubation. Determining the frequency of stem cells by using the extreme limit dilution analysis (ELDA) (http://bioinf.wehi.edu.au/software/elda/).

### Animal studies

All the animal experiments used in this research by the Chinese Ministry of Health were conducted in accordance with the Animal Control Regulations and according to the standards and experimental designs that Nanjing Medical University has authorized. Six-week-old nude mice (Charles River, Beijing, China) and pGBM-1 cell line were used to construct an orthotopic tumorigenesis model in nude mice. Two sets of nude mice were randomly assigned, and lentiviruses carrying the shcNDC80 and sh-CONT sequences were used to transplant pGBM-1 cells. To gauge the size of intracranial tumors, a total of 5 × 10^5^ pGBM-1 cells were stereotactically injected intracranially and regularly put in the imaging system. Nude mice were given an intraperitoneal injection of fluorescein potassium salt at a concentration of 50 mg/ml, given general anesthesia, and then put into an IVIS imaging system (Caliper Life Sciences, USA) for 10–120 s before to each experiment. The fluorescence intensity of the region where the cerebral tumor formed was measured and recorded using Living Images software (Caliper Life Sciences, USA).

### RNA immunoprecipitation (RIP) assay

For RNA-bound protein immunoprecipitation analysis, the Magna RIP kit (Millipore, USA) was obtained and used following the manufacturer’s guidelines. After lysing the cells in RIP lysis buffer, the lysates were treated with AGO2 (abcam ab32381) or IgG antibody magnetic beads and spun for an extended period at 4° C. The immunoprecipitated RNA was purified and normalized to the input control before being used to examine the cDNA using qRT–PCR.

### *CircRNA *in vivo* precipitation (circRIP) assay*

Using GenePharma (Shanghai, China), biotin-labeled circNDC80 probes were created and produced. In a 10-cm petri dish, cells were sown, and they were cultivated for 48 h at 37° C and 5% CO2. After that, cells were transfected for 24 h with either a particular biotin-labeled probe or a control probe (200 nM). The formaldehyde fixation process was then completed by equilibrating the cells with glycine for 5 min after they had been fixed for 10 min with 1% paraformaldehyde fixative. The cells were cleaned three times with precooled PBS, scraped clean with 1 ml of lysate. The samples underwent sonication (50% amplitude, continuous pulse for 30 s, halt for 30 s, and cycle for 10 min), 10000 *g* centrifugation, and the supernatant was transferred to a 2 mL centrifuge tube while 50uL was preserved as the Input control. The leftover supernatant was combined with magnetic beads that had been streptavidin-labeled by Invitrogen (USA) and let to sit at room temperature for the night. The mixture was then cleaned using lysis buffer containing proteinase K, and the crosslinking reaction was reversed. Enrichment levels were determined by qRT–PCR after total RNA was obtained with the miRNeasy mini kit (Qiagen, Germany).

### Dual-luciferase reporter assay

In order to create the luciferase reporter gene, the 3′untranslated region (3′UTR) sequence of circNDC80 or ECE1 containing the predicted and mutated binding sites was inserted into the vector pGL3. These constructs included circNDC80-WT, circNDC80-MUT, ECE1 3′UTR-WT, circNDC80-WT, circNDC80-MUT, and ECE1 3′UTR-WT and ECE1 3′UTR-MUT. After that, Lipofectamine 3000(Invitrogen, USA) was used to co-transfect the luciferase reporter gene with either miR-139-5p or miR-NC into cells. Finally, the dual-luciferase reporter gene assay kit (Vazyme, China) was employed in line with the instructions to measure the luciferase activity.

### Immunohistochemistry and Hematoxylin–eosin staining

The mouse brain was fixed with 4% paraformaldehyde (BOSTER, Wuhan, China), embedded in paraffin, and cut into 3.5–4 µm thick sections. Section stained with antibody (N-cadherin, Vimentin and SOX2). Brain tissue slices in paraffin blocks were extracted in xylene and hydrated in alcohol and distilled water before being stained with HE. The samples were then stained with hematoxylin (Sigma, USA) for 5 min after being rinsed three times with PBS for 5 min each. Eosin (USA, Sigma) was used to stain sections for 2 min so that researchers could see how clear the cytoplasm and nuclei were under a microscope. Images were examined and gathered under a microscope after regular dehydration and sealing.

### Statistical analysis

Three times each were done for every experiment. The program GraphPad Prism 9(La Jolla, USA) was used to plot all data analysis, which were all carried out using SPSS 17.0 (SPSS, USA) tool. A two-tailed Student’s *t*-test was utilized to determine if the distinctions between the two groups were statistically significant. The connection between circNDC80 and miR-139-5p was examined using the Spearman’s test. The Kaplan–Meier method was used to estimate overall survival.

## Results

### cNDC80 in gliomas: characterization and expression.

**Fig. 1:**
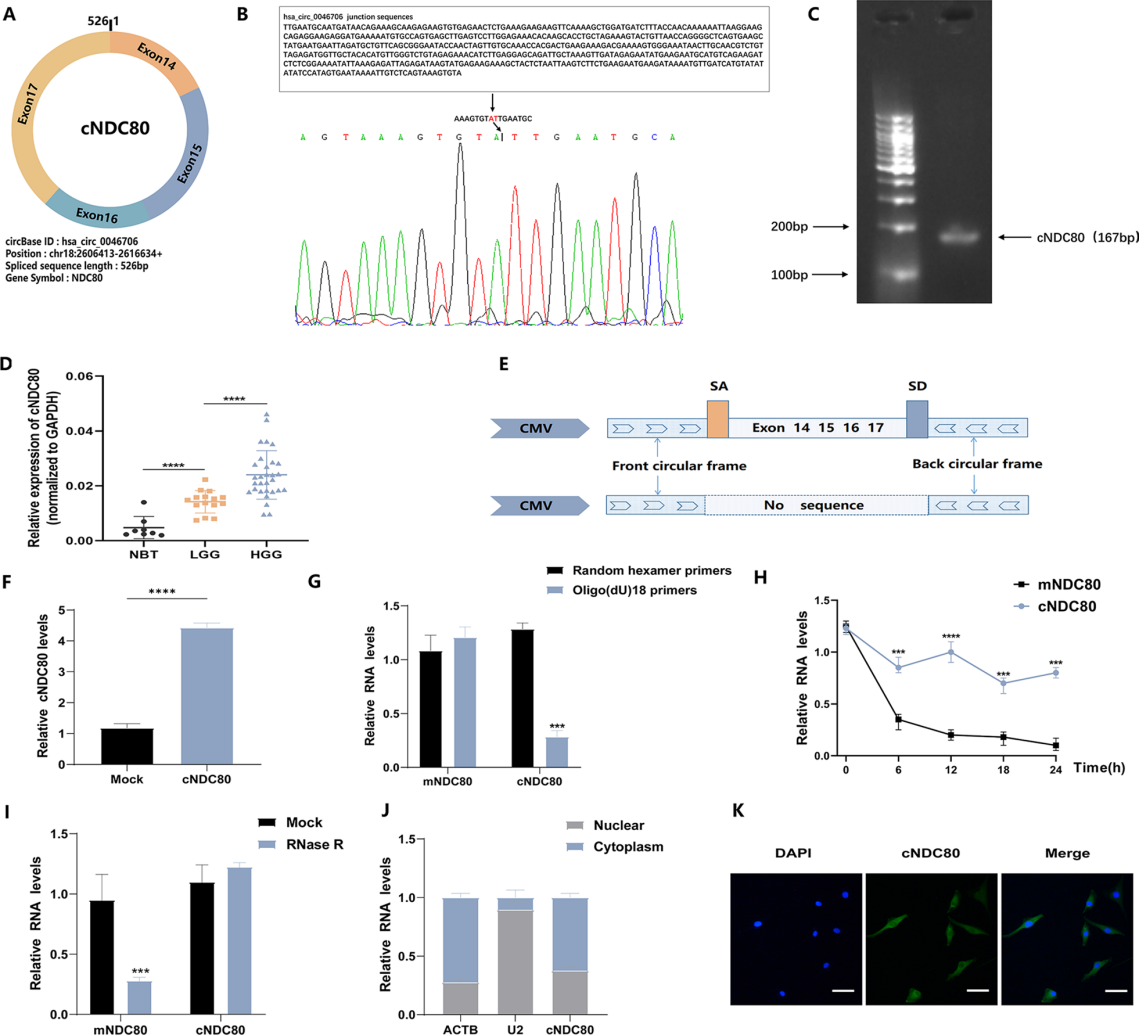
cNDC80 in gliomas: characterization and expression. **A** The splicing pattern and genomic location of the gene hsa_circ_0046706(526 bp) are shown in a schematic figure. **B** Sanger sequencing provides a schematic depiction of cNDC80’s structure. **C** The cNDC80 primers (167 bp) were verified using agarose gel electrophoresis. The arrow represents the “head to tail” splicing sites of cNDC80. **D** Eight normal brain tissues (NBTs), fifteen low-grade gliomas (LGGs), and thirty high-grade gliomas (HGGs) were analyzed for cNDC80 expression using qRT–PCR. **E** Construction of the cNDC80 and MOCK plasmid is shown schematically. **F** qRT–PCR was used to assess cNDC80 expression in U87 cells. **G** Primers such as oligo (dT)18 or random hexamer were used for the reverse transcription experiments. The relative RNA levels were determined by RT-qPCR using random hexamer primers as a standard. **H** The relative RNA levels in U87 cells were assessed by qRT–PCR at the given time periods after actinomycin D treatment. **I** After being treated with RNase R or a mock, the relative RNA levels in total RNAs obtained from U87 cells were assessed by qRT–qPCR. **J** Cellular RNA fractionation assays analyzed the cellular distribution of cNDC80. As cytoplasmic and nuclear positive controls, ACTB and U2 were utilized, respectively. **K** Fluorescence in situ hybridization (FISH) was used to investigate the cellular distribution of cNDC80. Green denotes the cNDC80. DAPI was applied to stain the nuclear. Scale bar, 100 μm. Each experiment was conducted three times, and the findings are shown as mean ± SD. (***P < 0.001, ****P < 0.0001)

Bioinformatics analysis revealed that cNDC80, a new circRNA that has not been previously investigated in glioma, is generated by circularization of exons 14 to 17 of the NDC80 gene. Mature cNDC80 has a length of 526 bp after splicing (Fig. 1A). We initially created a unique PCR primer for cNDC80 to measure cNDC80 expression in glioma tissue. The construct was confirmed by Sanger sequencing and agarose gel electrophoresis (Fig. 1B and C). Then, using qRT‒PCR, we discovered that thirty high-grade glioma (HGG) tissues had considerably higher levels of cNDC80 expression than fifteen low-grade glioma (LGG) tissues and eight normal brain tissues (NBTs) (Fig. 1D).

Then, we carried out several tests to validate the circular RNA properties of cNDC80. First, we created cNDC80 and mock expression vectors, and then we performed qRT‒PCR in GBM cells (U87) to confirm the effectiveness of the cNDC80 overexpression vectors (Fig. 1E and F). Additionally, utilizing total RNA from U87 cells, we performed reverse transcription using oligo(dT)18 primers or random hexamers. In comparison to that obtained with the random hexamer primer, the relative expression of cNDC80 with the oligo(dT)18 primer was much lower. However, in contrast, the expression of mature NDC80 mRNA (mNDC80) remained unchanged, and the experimental results demonstrated that cNDC80 had no poly(-A)-tail (Fig. 1G). In addition, we found that cNDC80 was more stable than mNDC80 by measuring the half-lives of cNDC80 and mNDC80 after treatment with the transcription inhibitor actinomycin D in pGBM-1 cell lines (Fig. 1H). Previous research has demonstrated that circRNAs are resistant to RNase R digestion and more stable than linear RNAs; hence, RNase R was used to degrade total RNA. The results showed that cNDC80 was more stable upon exposure to RNase R than linear NDC80 mRNA. (Fig. 1I) Utilizing cellular RNA fractionation, we also discovered that the majority of cNDC80 in GBM cells (U87) was localized in the cytoplasm (Fig. 1J) and FISH was also used to validate the findings (Fig. 1K).

These findings proved that cNDC80 is a circulating transcript that is stable.

### cNDC80 induces GBM cell proliferation, migration, and invasion in vitro

**Fig. 2:**
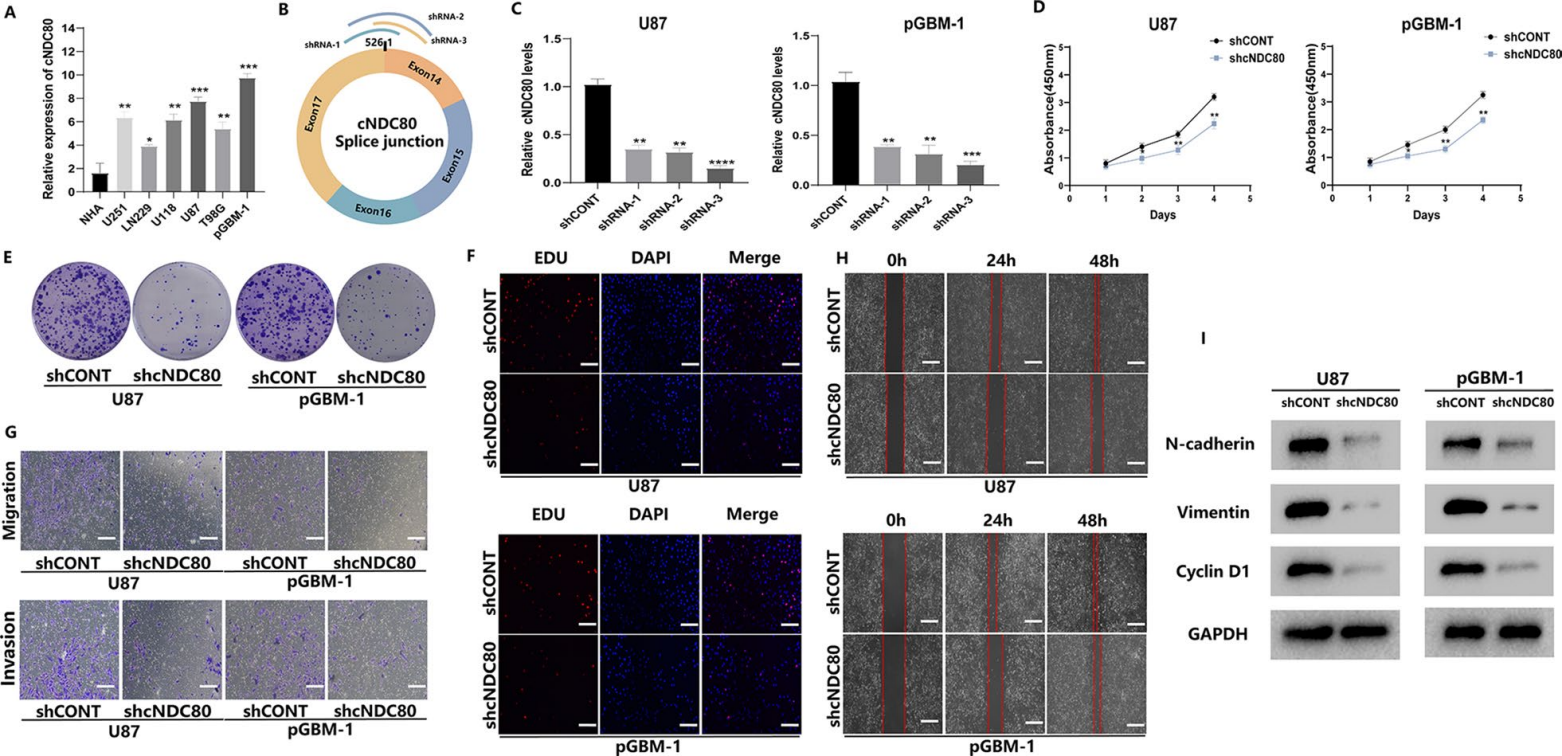
cNDC80 induces GBM cell proliferation, migration, and invasion in vitro. **A** The expression levels of cNDC80 in GBM cells and NHAs were investigated using qRT–PCR. **B** Designated shRNAs for cNDC80 are shown schematically at splice junctions. **C** In U87 and pGBM-1 cells transduced with either cNDC80-targeting shRNA-3 or a non-targeted control shRNA, the expression of cNDC80 was measured by qRT–PCR. **D**–**F** CCK-8, colony formation, and EdU were used to identify the cell proliferation capacities of transfected U87 and pGBM-1 cells. **G** Transwell for cell migration and invasion studies after expression vector or shRNA transfection of glioma cells. **H** Using the wound healing assay, the migration of GBM cells was evaluated. **I** Following transfection in GBM cells, N-cadherin, Vimentin, and CyclinD1 were analyzed using Western blots. Each experiment was conducted three times, and the findings are shown as mean ± SD. (*P < 0.05, **P < 0.01, ***P < 0.001, ****P < 0.0001)

We conducted qRT‒PCR to determine the expression of cNDC80 in a human glioblastoma primary cell line derived from an adult patient (pGBM-1), GBM cell lines (U251, LN229, U118, U87, T98) and NHAs. U87 and pGBM-1 cells expressed cNDC80 at a higher level than the other cell lines (Fig. 2A). Therefore, we used U87 and pGBM-1 cells to carry out subsequent experiments. First, for loss-of-function studies, we performed transduction of three separate lentiviral vectors expressing short hairpin RNAs (shRNAs) that targeted cNDC80, called shRNA-1, shRNA-2, and shRNA-3 (Fig. 2B). Real-time PCR results supporting the effectiveness of cNDC80 knockdown led to the selection of shRNA-3 (shcNDC80) for further research (Fig. 2C). Silencing cNDC80 expression decreased the proliferation of U87 and pGBM-1 cells, according to observations from the CCK-8 assay, clone formation assay, and EdU incorporation assay (Fig. 2D–F and Additional file 1: Fig. S1A, B). Additionally, the Transwell and wound healing assays demonstrated that U87 and pGBM-1 cell migration and invasion were reduced when cNDC80 expression was silenced (Fig. 2G, H and Additional file 1: Fig. S2C, D). Silencing cNDC80 significantly reduced the expression levels of migration, invasion, and proliferation markers (N-cadherin, Vimentin, and Cyclin D1) (Fig. 2I). We also demonstrated using immunofluorescence staining that silencing cNDC80 dramatically decreased the expression levels of N-cadherin and Vimentin in transduced glioma cells (Additional file 1: Fig. S1E).

### cNDC80 promotes GSC maintenance in vitro

**Fig. 3:**
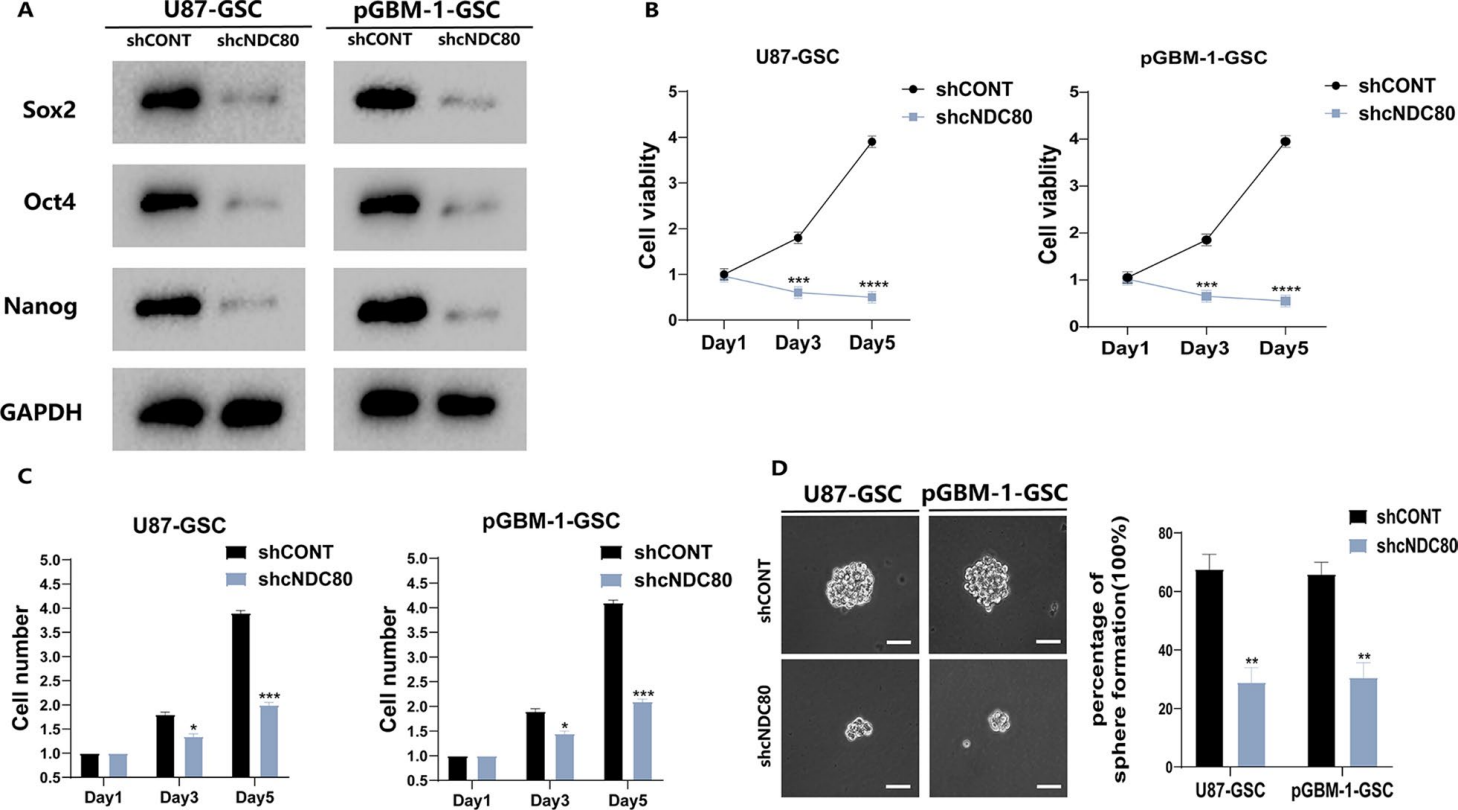
cNDC80 enhanced GSC maintenance in vitro. **A** Following transfection in GBM–GSC cells, Sox2, Oct4, and Nanog were analyzed using Western blots. **B** CellTiter-Glo assay was utilized to evaluate the cellular impacts in GBM–GSC cells. **C** The impact of cNDC80 knockdown on the growth of GBM–GSC cells was determined by direct cell counting. **D** After transfection, GSC stemness was assessed using a clonogenic test. Typical photomicrographs of the newly formed clonal sphere (left) and statistical analysis of the percentage of positive wells (right). Scale bar, 100 µm. Each experiment was conducted three times, and the findings are shown as mean ± SD. (*P < 0.05, **P < 0.01, ***P < 0.001, ****P < 0.0001)

The growth and viability of GSC cells (U87 and pGBM-1) were impacted by cNDC80. The growth and maintenance of GSCs depend on the reprogramming transcription factors Sox2, Oct4 and Nanog, which serve as key multipotency factors. Next, Western blotting was used to detect the pluripotency factors Sox2, Oct4, and Nanog (Fig. 3A). GSC viability and growth were decreased when cNDC80 was knocked down (Fig. 3B–D and Additional file 2: Fig. S2A), thus proving that cNDC80 is a critical factor in regulating GSC maintenance.

### Knockdown of cNDC80 inhibits GBM growth in vivo

**Fig. 4:**
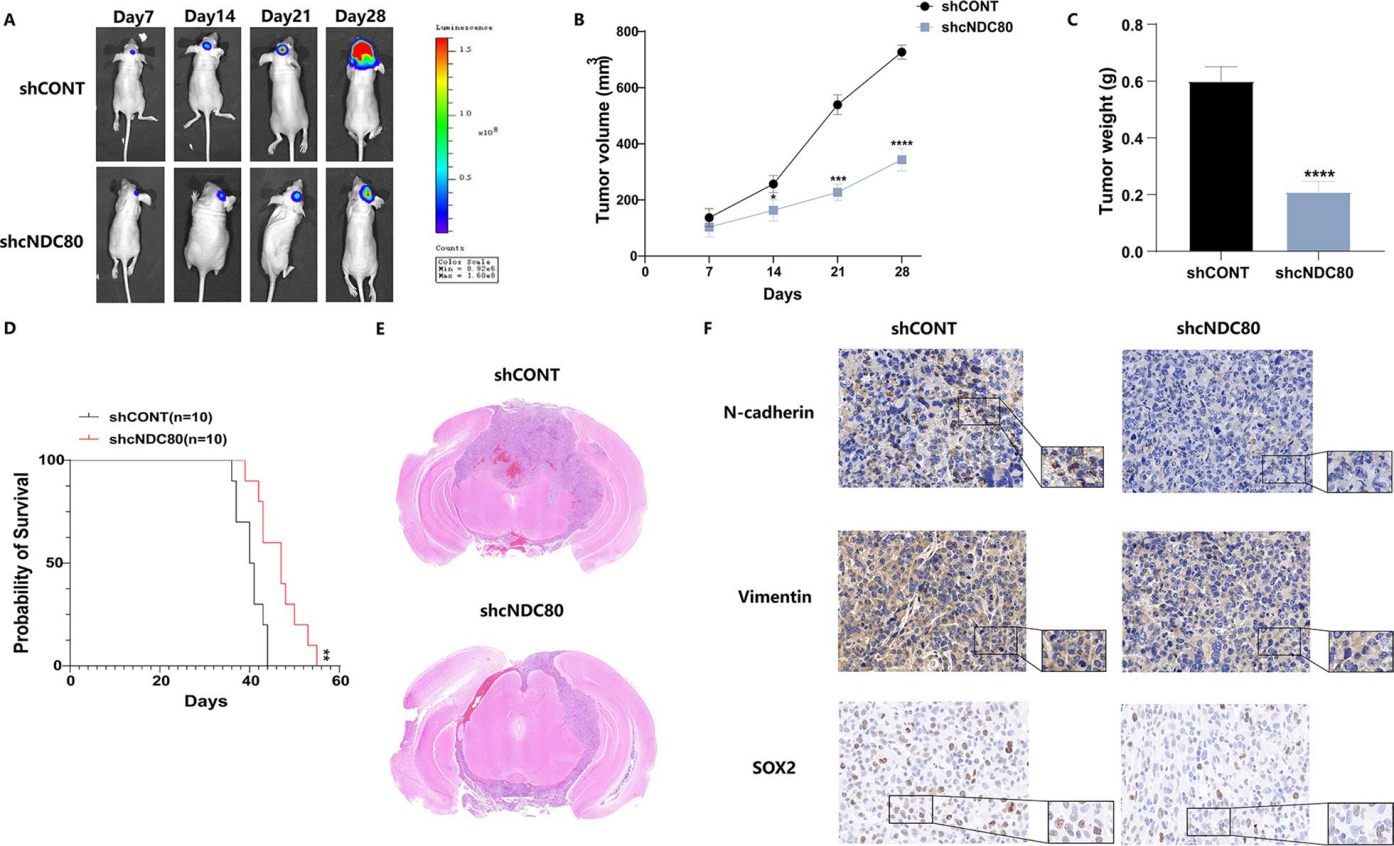
Knockdown of cNDC80 inhibits GBM growth in vivo*.* **A**–**C** After stereotaxic orthotopic implantation of glioma cells weekly, bioluminescent pictures of the intracerebral tumors of nude mice (10 mice per group) were obtained. **D** Kaplan–Meier survival analysis revealed that mice in the shcNDC80 group outlived those in the shCONT group. **E** The tumor volume of the shcNDC80 group was dramatically decreased, according to HE histology. **F** Tumors grown as xenografts in the shCONT and shcNDC80 groups were analyzed using immunohistochemistry. Scale bar 100um. Each experiment was conducted three times, and the findings are shown as mean ± SD. (*P < 0.05, **P < 0.01, ***P < 0.001, ****P < 0.0001)

To gain more insight into how cNDC80 affects glioma cells in vivo, an intracranial xenograft tumour model was established. Stereotaxic injection of shCONT or shcNDC80-transfected pGBM-1 cells into the brain was carried out. After implanting intracranial xenografts of pGBM-1 cells in nude mice, we employed weekly bioluminescence imaging to track tumour growth. The inhibition of intracranial tumour development by cNDC80 silencing was considerable (observed on Days 7, 14, 21, and 28) (Fig. 4A–C). Mice injected with shcNDC80 survived longer than mice injected with shCONT, as shown by the Kaplan–Meier survival curves (Fig. 4D). HE staining was used concurrently to measure the tumour volume in mice and the tumour volume in the shcNDC80 group was dramatically decreased (Fig. 4E). Additionally, immunohistochemical analysis confirmed the results of in vitro investigations by demonstrating that the expression of N-cadherin, Vimentin and SOX2 was reduced in specimens of derived from shcNDC80 pGBM-1 cells. (Fig. 4F).

### cNDC80 functions as a sponge for miR-139-5p

**Fig. 5:**
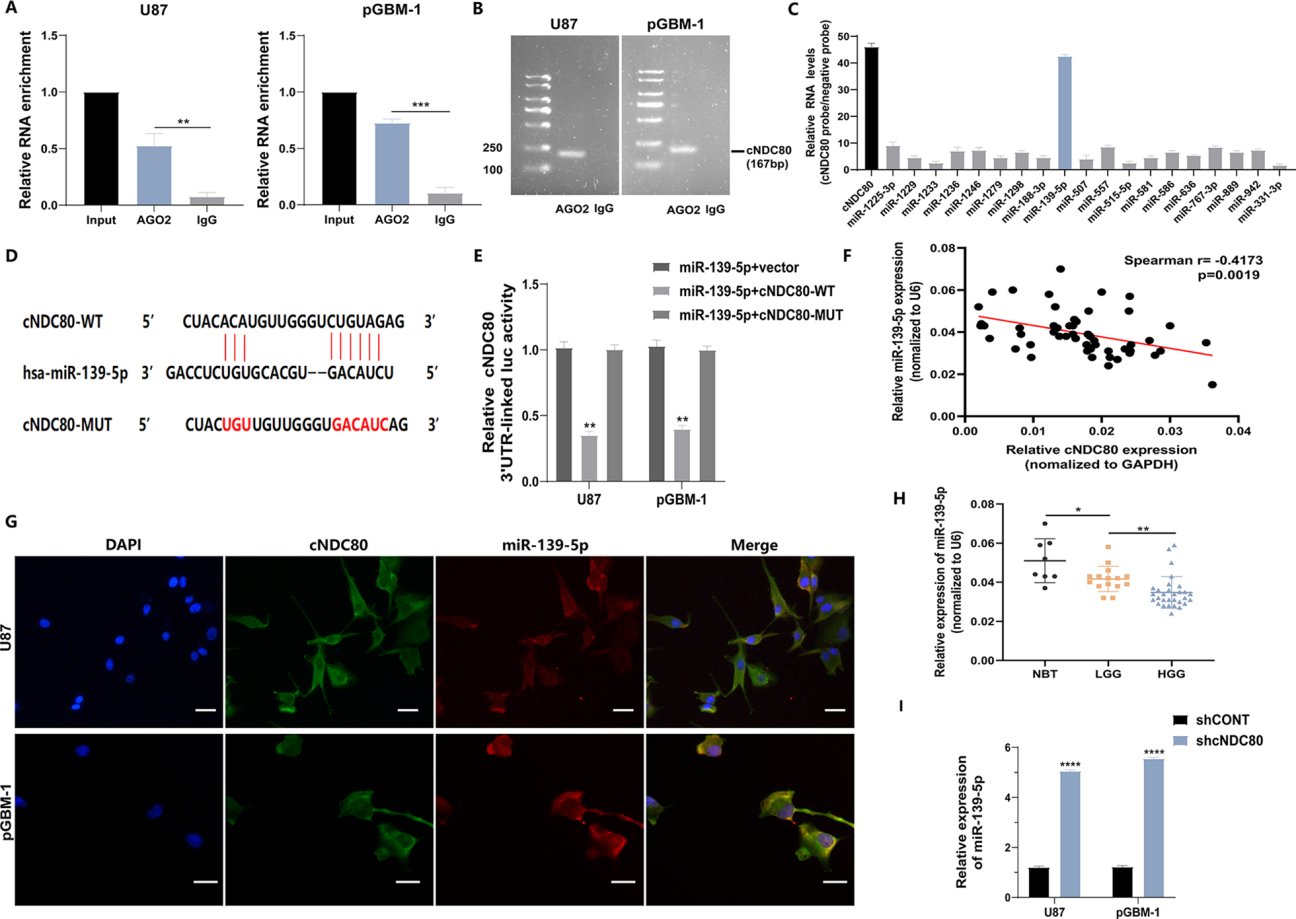
cNDC80 functions as a sponge for miR-139-5p. **A** Evaluation of cNDC80 binding to the AGO2 protein was performed using RNA immunoprecipitation (RIP) and RT-qPCR techniques. **B** Testing on agarose gel electrophoresis confirmed that cNDC80 binds to the AGO2 proteins. The cDNA for cNDC80 is 167 base pairs long. **C** cNDC80-overexpressing U87 cells were used in combination with a cNDC80-specific probe and an NC probe for circRNA in vivo precipitation (circRIP) experiments. Potential miRNAs connected to cNDC80 were examined using RT-qPCR assays. The enrichment of cNDC80 and putative miRNAs was normalized by comparing it to the control probe. **D** Possible binding site sequences between cNDC80 and miR-139-5p, both in the wild-type (WT) and mutated-type (MUT). **E** Dual-luciferase reporter assays were carried out to identify the relationship between cNDC80 and miR-139-5p. **F** The connection between cNDC80 and miR-139-5p expression levels was analyzed using Pearson’s correlation (r = − 0.4173, P = 0.0019). **G** FISH was applied to examine the colocalization of cNDC80 and miR-139-5p in transfected GBM cells. Scar bar 100 um. **H** qRT–PCR was applied to examine at the expression of miR-139-5p in thirty high-grade gliomas, fifteen low-grade gliomas, and eight normal brain tissues. **I** In treated GBM cells, qRT–PCR was used to verify the expression of miR-139-5p. Each experiment was conducted three times, and the findings are shown as mean ± SD. (*P < 0.05, **P < 0.01, ***P < 0.001, ****P < 0.0001)

Previous studies have shown that circRNAs primarily function as sponges for miRNAs to regulate gene expression. Using an anti-AGO2 antibody, RNA immunoprecipitation (RIP) assays were performed to determine whether cNDC80 functions as a miRNA sponge in glioma cells. The results of qRT‒PCR and agarose gel electrophoresis showed that cNDC80 enrichment was significantly increased in the anti-AGO2 precipitate. In contrast, cNDC80 enrichment was significantly decreased in the IgG precipitate (Fig. 5A and B), indicating that cNDC80 could bind to AGO2 and miRNAs. Potential miRNA targets of cNDC80 were predicted using the Circular RNA Interactome Database. The results of the circRIP assay indicated that cNDC80 and miR-139-5p were more enriched by the cNDC80-specific probe than were the other miRNAs, indicating that cNDC80 and miR-139-5p may be targets in GBM cells (Fig. 5C).

The putative binding sites of miR-139-5p and cNDC80 were used to generate dual luciferase reporter vectors containing wild-type (WT) and mutant (MUT) of cNDC80 sequences to further confirm this finding (Fig. 5D). The results showed that transfection of the miR-139-5p mimic significantly reduced luciferase activity compared to that in cells transfected with the mutant cNDC80 vector (Fig. 5E). Additionally, the cNDC80 and miR-139-5p expression levels in glioma samples were shown to be negatively correlated by Spearman correlation analysis (Fig. 5F). cNDC80 and miR-139-5p were also shown to colocalize in the cytoplasm, based on immunofluorescence analysis (Fig. 5G). The expression level of miR-139-5p in glioma tissues was significantly lower than that in NBTs, as determined by qPCR (Fig. 5H). Downregulation of cNDC80 significantly increased the expression of miR-139-5p in U87 and pGBM-1 cells (Fig. 5I). From the above analyses, it seems that in GBM cells, cNDC80 may act as a sponge for miR-139-5p.

### ECE1 is a direct target of miR-139-5p

**Fig. 6:**
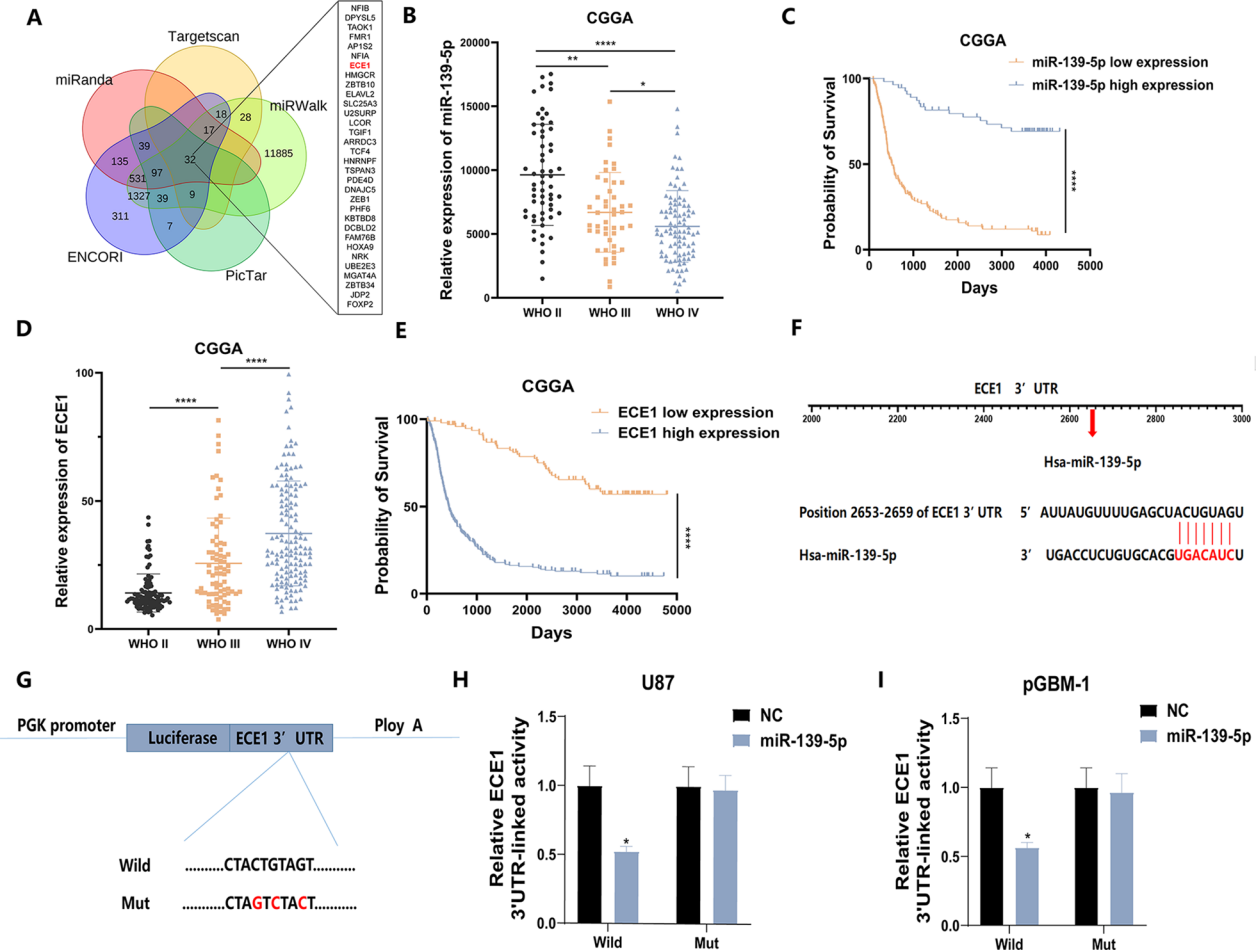
ECE1 is a direct target of miR-139-5p. **A** Using a Venn diagram, it can be shown that miR-139-5p may have 32 mRNA as biological targets. **B** The expression of miR-139-5p in the glioma patient databank housed in CGGA database. **C** Using the CGGA database, survival rates were compared between two groups of patients defined by their miR-139-5p levels using the Kaplan–Meier method. **D** Analyzing ECE1 expression in the CGGA database. **E** Using the CGGA database, we divided patients with regards to their ECE1 levels and created a Kaplan–Meier survival curve to compare the survival rates between the two groups. **F** The putative binding sites of miR-139-5p on ECE1. **G** ECE1 3′UTR reporter constructions, both wild-type and mutant. **H**, **I** In GBM cells, the functional relationship between miR-139-5p and ECE1 was confirmed using a luciferase reporter experiment. Each experiment was conducted three times, and the findings are shown as mean ± SD. (*P < 0.05, **P < 0.01, ***P < 0.001, ****P < 0.0001)

We next sought to verify that ECE1 is a possible target of miR-139-5p by using the bioinformatics databases miRanda, Targetscan, PicTar, miRWalk, and ENCORI (Fig. 6A). To investigate the role and significance of miR-139-5p in glioma, the expression of miR-139-5p and its relationship to clinical prognosis were analysed using the CGGA (China Glioma Genome Atlas) database. The expression of miR-139-5p significantly decreased as glioma grade increased (Fig. 6B). The survival times of patients with high expression of miR-139-5p were longer (Fig. 6C). Analysis of the CGGA data indicated that as the glioma grade increased, the expression of ECE1 also increased (Fig. 6D). The survival times of patients with low expression of ECE1 were longer (Fig. 6E). Previous studies have shown that miRNAs inhibit mRNA transcription or induce mRNA degradation by binding to their target mRNA molecules' 3′ -UTRs. Thus, we assumed that miR-139-5p binds to the 3′ -UTRs of ECE1 to control the development of gliomas (Fig. 6F). To determine whether miR-139-5p can directly bind to the 3′UTR of ECE1, a luciferase reporter assay was carried out. To this end, the wild-type or mutant ECE1 3′UTR was cotransfected with the miR-139-5p mimic (Fig. 6G), and in U87 and pGBM-1 cells cotransfected with the miR-139-5p mimic mutation of the binding site entirely reversed the trend of decreasing activity. (Fig. 6H and I).

### cNDC80 enhanced GBM cell proliferation, migration, and invasion, and GSC maintenance through the miR-139-5p/ECE1 pathway

**Fig. 7:**
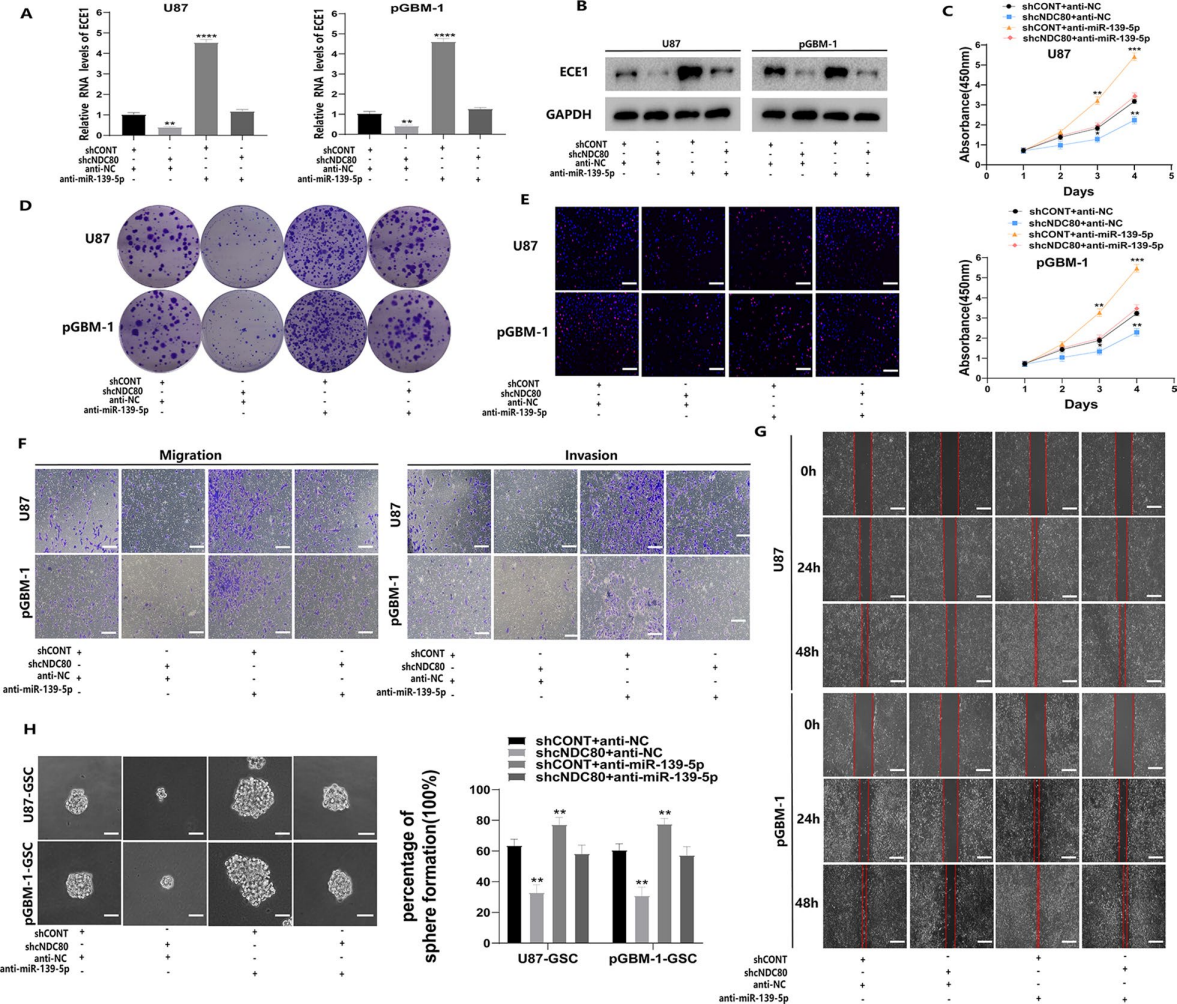
cNDC80 enhanced GBM cell proliferation, migration, and invasion, and GSC maintenance through the miR-139-5p/ECE1 pathway. **A** By using qRT–PCR assays, to determine the expression levels of ECE1 in GBM cells after silencing miR-139-5p alone or in conjunction with the silencing of cNDC80. **B** ECE1 was analyzed using a Western blot in the specified cells, with GPAPDH acting as a control. **C**–**E** GBM cells transfected with sh-cNDC80, anti-miR-139-5p, or anti-miR-139-5p in addition to sh-cNDC80 were examined for proliferation employing the CCK-8 test, the clone formation assay, and the EdU assay. **F** GBM cells were transfected with sh-cNDC80, anti-miR-139-5p, or anti-miR-139-5p and sh-cNDC80 to study the migration and invasion of the cells by Transwell. **G** U87 and pGBM-1 cells transfected with sh-cNDC80, anti-miR-139-5p, or anti-miR-139-5p and sh-cNDC80 were examined utilizing the Wound healing test for migration and invasion. **H** The stemness of GSCs after transduction was assessed by analyzing the percentage of positive wells in a clonogenic assay and by analyzing typical photomicrographs of the new clonal sphere. Each experiment was conducted three times, and the findings are shown as mean ± SD. (**P < 0.01, ***P < 0.001, ****P < 0.0001)

Then, we examined the relationship between cNDC80 and miR-139-5p and how it affects the expression of ECE1, the malignant behaviour of GBM cells, and the survival of GSCs. Rescue experiments were performed to further verify the integrity of the ceRNA pathway. Knockdown of cNDC80 markedly reduced ECE1 mRNA and protein levels. At the same time, if we further inhibited the expression of miR-139-5p in cells with cNDC80 silencing, the effect caused by cNDC80 silencing was reversed (Fig. 7A). Subsequently, we further verified the results by Western blotting (Fig. 7B). Regarding the functional experiments, CCK-8 assays, clone formation assays, and EdU incorporation assays confirmed that cNDC80 downregulation inhibited the proliferation of GBM cells, and that miR-139-5p inhibitor treatment reversed the effects of cNDC80 silencing (Fig. 7C–E and Additional file 3: Fig. S3A, B). The transwell and wound healing assay results supported the finding that downregulating cNDC80 prevented GBM cells from migrating and invading. Similarly, the impact of cNDC80 silencing on GBM cells may be reversed by miR-139-5p inhibitor treatment. (Fig. 7F–G and Additional file 3: Fig. S3C, D). In addition, inhibition of miR-139-5p blocked the inhibitory effect of cNDC80 on GSC maintenance and growth (Fig. 7H and Additional file 3: Fig. S3E–G). These findings showed that cNDC80 promoted the development of gliomas by regulating ECE1 expression by serving as a sponge for miR-139-5p.

## Discussion

The most common and malignant histologic type of glioma is glioblastoma (GBM) [33, 34]. GBM is difficult to entirely resect by surgery and has a clear tolerance to radiotherapy [35, 36]. At the same time, it has high recurrence rates and mortality rate because of its rapid proliferation and ability to infiltrate and invade, which are reported to be related to glioma stem cells (GSCs) [37–39].

CircRNA is exceptionally stable in biological cells and is a popular target for treating human diseases [40]. CircRNA transcription was discovered as early as the 1990s. However, the related research was limited by the technology and knowledge level at that time, and scientific researchers could not conduct sufficient and detailed research [41]. Recent research has shown that circRNAs are crucial to the development of glioma [42, 43]. In non-small cell lung adenocarcinoma cells, NDC80 activates cancer stem cell characteristics. NDC80 independently predicts survival, but its role in glioma is unknown [44]. Therefore, we hypothesized that circNDC80 promotes the biological progression of GBM and the stem cell-like phenotype of glioma cells. We discovered that cNDC80 was extremely highly expressed in glioma samples, and that silencing cNDC80 suppressed the malignant expression patterns in glioma stem cells (GSCs), and the invasion, migration, and proliferation of GBM cells in vitro. There are certain limitations, however, as we only ran qRT‒PCR on a small number of samples. To validate the carcinogenic impact of cNDC80, we need to test additional GBM tissues in future research.

CircRNAs act as a miRNA sponges and promote the growth of gliomas, according to previous studies [45]. First, we predicted the possible miRNAs targeted by cNDC80 using bioinformatics tools. Then, to confirm that cNDC80 can bind to miR-139-5p, circRIP, dual-luciferase reporter, and colocalization assays were applied. Moreover, using different miRNA target gene prediction software programs, we found that the ECE1 mRNA may function as a downstream miRNA target that directly regulates GBM. Western blotting further demonstrated that miR-139-5p may counteract the considerable increase in ECE1 expression that resulted from cNDC80 knockdown in GBM cells. The information above demonstrates the oncogenic involvement of cNDC80 in the development of gliomas.

CircNDC80 may promote the proliferation and stemness maintenance of glioma cells, as well as the migration and invasion of glioma cells, which may be closely related to the rapid increase in tumour volume in a patient's brain, the degeneration of peritumoral tissues and postoperative recurrence.

## Conclusion

Our work concluded by demonstrating that circNDC80 expression was increased in glioma tissues compared to healthy brain tissues and that it enhanced GBM cell proliferation, migration, and invasion by sponging miR-139-5p while preserving the stemness of GSCs to enhance GSC self-renewal. The above factors make complete surgical resection of GBM difficult and cause GBM to be prone to recurrence. As a result, this novel discovery may help us better comprehend the molecular basis of glioblastoma and provide creative solutions for glioma diagnosis and treatment.

## Supplementary Information

**Additional file 1: Figure S1. **(A) Statistical bar chart of U87 and pGBM-1 clone formation. (B) Statistical bar chart of EDU positive rate of U87 and pGBM-1. (C-D) Transwell statistical bar chart of U87 and pGBM-1. (E)N-cadherin and Vimentin immunofluorescence staining of GBM cells after treatment. Scale bar, 100 µm. Each experiment was conducted three times, and the findings are shown as mean ± SD. (*P < 0.05, **P < 0.01, ***P < 0.001, ****P < 0.0001).

**Additional file 2: Figure S2.** (A) The capacity of GSCs to proliferate was discovered using the extreme limit dilution test. Each experiment was conducted three times, and the findings are shown as mean ± SD. (*P < 0.05, **P < 0.01, ***P < 0.001, ****P < 0.0001).

**Additional file 3: Figure S3.** (A) Statistical bar chart of U87 and pGBM-1 clone formation. (B) Statistical bar chart of EDU positive rate of U87 and pGBM-1. (C-D) Transwell statistical bar chart of U87 and pGBM-1. (E-F) A direct cell count and CellTiter-Glo assay were used to examine the growth and cellular effects of transduced U87-GSCs and pGBM-1-GSCs. (G) The capacity of GSCs to proliferate was discovered using the extreme limit dilution test. Each experiment was conducted three times, and the findings are shown as mean ± SD. (*P < 0.05, **P < 0.01, ***P < 0.001, ****P < 0.0001).

**Additional file 4: Table S1.** Primers used in the present study.

**Additional file 5: Table S2.** Patient information and diagnostic criteria.

## Data Availability

On reasonable request, the corresponding author will provide the datasets used and/or analyzed during the current study.

## References

[1] Cordier D, Krolicki L, Morgenstern A, Merlo A (2016). Targeted radiolabeled compounds in glioma therapy. Semin Nucl Med.

[2] Chen R, Smith-Cohn M, Cohen AL, Colman H (2017). Glioma subclassifications and their clinical significance. Neurotherapeutics.

[3] Wesseling P, Capper D (2018). WHO 2016 classification of gliomas. Neuropathol Appl Neurobiol.

[4] Bilmin K, Kujawska T, Grieb P (2019). Sonodynamic therapy for gliomas. Perspectives and prospects of selective sonosensitization of glioma cells. Cells.

[5] Hervey-Jumper SL, Berger MS (2019). Insular glioma surgery: an evolution of thought and practice. J Neurosurg.

[6] Ohgaki H, Kleihues P (2005). Epidemiology and etiology of gliomas. Acta Neuropathol.

[7] Xu S, Tang L, Li X, Fan F, Liu Z (2020). Immunotherapy for glioma: current management and future application. Cancer Lett.

[8] Beck B, Blanpain C (2013). Unravelling cancer stem cell potential. Nat Rev Cancer.

[9] Vlashi E, Pajonk F (2015). Cancer stem cells, cancer cell plasticity and radiation therapy. Semin Cancer Biol.

[10] Batlle E, Clevers H (2017). Cancer stem cells revisited. Nat Med.

[11] Bao S, Wu Q, McLendon RE, Hao Y, Shi Q, Hjelmeland AB, Dewhirst MW, Bigner DD, Rich JN (2006). Glioma stem cells promote radioresistance by preferential activation of the DNA damage response. Nature.

[12] Fan X, Salford LG, Widegren B (2007). Glioma stem cells: evidence and limitation. Semin Cancer Biol.

[13] Stiles CD, Rowitch DH (2008). Glioma stem cells: a midterm exam. Neuron.

[14] Bexell D, Svensson A, Bengzon J (2013). Stem cell-based therapy for malignant glioma. Cancer Treat Rev.

[15] Chen L, Shan G (2021). CircRNA in cancer: fundamental mechanism and clinical potential. Cancer Lett.

[16] Li R, Jiang J, Shi H, Qian H, Zhang X, Xu W (2020). CircRNA: a rising star in gastric cancer. Cell Mol Life Sci.

[17] Yang Y, Yujiao W, Fang W, Linhui Y, Ziqi G, Zhichen W, Zirui W, Shengwang W (2020). The roles of miRNA, lncRNA and circRNA in the development of osteoporosis. Biol Res.

[18] Kristensen LS, Andersen MS, Stagsted LVW, Ebbesen KK, Hansen TB, Kjems J (2019). The biogenesis, biology and characterization of circular RNAs. Nat Rev Genet.

[19] Sang Y, Chen B, Song X, Li Y, Liang Y, Han D, Zhang N, Zhang H, Liu Y, Chen T (2019). circRNA_0025202 regulates tamoxifen sensitivity and tumor progression via regulating the miR-182-5p/FOXO3a axis in breast cancer. Mol Ther.

[20] Zheng X, Huang M, Xing L, Yang R, Wang X, Jiang R, Zhang L, Chen J (2020). The circRNA circSEPT9 mediated by E2F1 and EIF4A3 facilitates the carcinogenesis and development of triple-negative breast cancer. Mol Cancer.

[21] Huang G, Liang M, Liu H, Huang J, Li P, Wang C, Zhang Y, Lin Y, Jiang X (2020). CircRNA hsa_circRNA_104348 promotes hepatocellular carcinoma progression through modulating miR-187-3p/RTKN2 axis and activating Wnt/beta-catenin pathway. Cell Death Dis.

[22] Chen D, Ma W, Ke Z, Xie F (2018). CircRNA hsa_circ_100395 regulates miR-1228/TCF21 pathway to inhibit lung cancer progression. Cell Cycle.

[23] Qi X, Zhang D-H, Wu N, Xiao J-H, Wang X, Ma W (2015). ceRNA in cancer: possible functions and clinical implications. J Med Genet.

[24] Lu TX, Rothenberg ME (2018). MicroRNA. J Allergy Clin Immunol.

[25] Saliminejad K, Khorram Khorshid HR, Soleymani Fard S, Ghaffari SH (2019). An overview of microRNAs: biology, functions, therapeutics, and analysis methods. J Cell Physiol.

[26] Hong Y, Macnab S, Lambert LA, Turner AJ, Whitehouse A, Usmani BA (2011). Herpesvirus saimiri-based endothelin-converting enzyme-1 shRNA expression decreases prostate cancer cell invasion and migration. Int J Cancer.

[27] Tapia JC, Niechi I (2019). Endothelin-converting enzyme-1 in cancer aggressiveness. Cancer Lett.

[28] Chu H, Duan Y, Lang S, Jiang L, Wang Y, Llorente C, Liu J, Mogavero S, Bosques-Padilla F, Abraldes JG (2020). The *Candida*
*albicans* exotoxin candidalysin promotes alcohol-associated liver disease. J Hepatol.

[29] Engku Nasrullah Satiman EA, Ahmad H, Ramzi AB, Abdul Wahab R, Kaderi MA, Wan Harun WHA, Dashper S, McCullough M, Arzmi MH (2020). The role of *Candida*
*albicans* candidalysin ECE1 gene in oral carcinogenesis. J Oral Pathol Med.

[30] Perez-Moreno P, Indo S, Niechi I, Huerta H, Cabello P, Jara L, Aguayo F, Varas-Godoy M, Burzio VA, Tapia JC (2020). Endothelin-converting enzyme-1c promotes stem cell traits and aggressiveness in colorectal cancer cells. Mol Oncol.

[31] Liu L, Zhang P, Dong X, Li H, Li S, Cheng S, Yuan J, Yang X, Qian Z, Dong J (2021). Circ_0001367 inhibits glioma proliferation, migration and invasion by sponging miR-431 and thus regulating NRXN3. Cell Death Dis.

[32] Zhou M, Yang Z, Wang D, Chen P, Zhang Y (2021). The circular RNA circZFR phosphorylates Rb promoting cervical cancer progression by regulating the SSBP1/CDK2/cyclin E1 complex. J Exp Clin Cancer Res.

[33] Ostrom QT, Bauchet L, Davis FG, Deltour I, Fisher JL, Langer CE, Pekmezci M, Schwartzbaum JA, Turner MC, Walsh KM (2014). The epidemiology of glioma in adults: a “state of the science” review. Neuro Oncol.

[34] Eisele SC, Reardon DA (2016). Adult brainstem gliomas. Cancer.

[35] Krivosheya D, Prabhu SS, Weinberg JS, Sawaya R (2016). Technical principles in glioma surgery and preoperative considerations. J Neurooncol.

[36] Thomas JG, Parker Kerrigan BC, Hossain A, Gumin J, Shinojima N, Nwajei F, Ezhilarasan R, Love P, Sulman EP, Lang FF (2018). Ionizing radiation augments glioma tropism of mesenchymal stem cells. J Neurosurg.

[37] Gunther HS, Schmidt NO, Phillips HS, Kemming D, Kharbanda S, Soriano R, Modrusan Z, Meissner H, Westphal M, Lamszus K (2008). Glioblastoma-derived stem cell-enriched cultures form distinct subgroups according to molecular and phenotypic criteria. Oncogene.

[38] Sampetrean O, Saya H (2013). Characteristics of glioma stem cells. Brain Tumor Pathol.

[39] Wang X, Zhou R, Xiong Y, Zhou L, Yan X, Wang M, Li F, Xie C, Zhang Y, Huang Z (2021). Sequential fate-switches in stem-like cells drive the tumorigenic trajectory from human neural stem cells to malignant glioma. Cell Res.

[40] Zhou WY, Cai ZR, Liu J, Wang DS, Ju HQ, Xu RH (2020). Circular RNA: metabolism, functions and interactions with proteins. Mol Cancer.

[41] Patop IL, Wust S, Kadener S (2019). Past, present, and future of circRNAs. EMBO J.

[42] Cheng J, Meng J, Zhu L, Peng Y (2020). Exosomal noncoding RNAs in Glioma: biological functions and potential clinical applications. Mol Cancer.

[43] Sun J, Li B, Shu C, Ma Q, Wang J (2020). Functions and clinical significance of circular RNAs in glioma. Mol Cancer.

[44] Chen J, Chen H, Yang H, Dai H (2018). SPC25 upregulation increases cancer stem cell properties in non-small cell lung adenocarcinoma cells and independently predicts poor survival. Biomed Pharmacother.

[45] Zhang J, Liu Y, Shi G (2021). The circRNA-miRNA-mRNA regulatory network in systemic lupus erythematosus. Clin Rheumatol.

