# Supplementary figures and images for: CircNDC80 promotes glioblastoma multiforme tumorigenesis via the miR-139-5p/ECE1 pathway
Source: J Transl Med. 2023 Jan 12;21:22. doi: 10.1186/s12967-022-03852-3 (PMC9837923; doi:10.1186/s12967-022-03852-3)

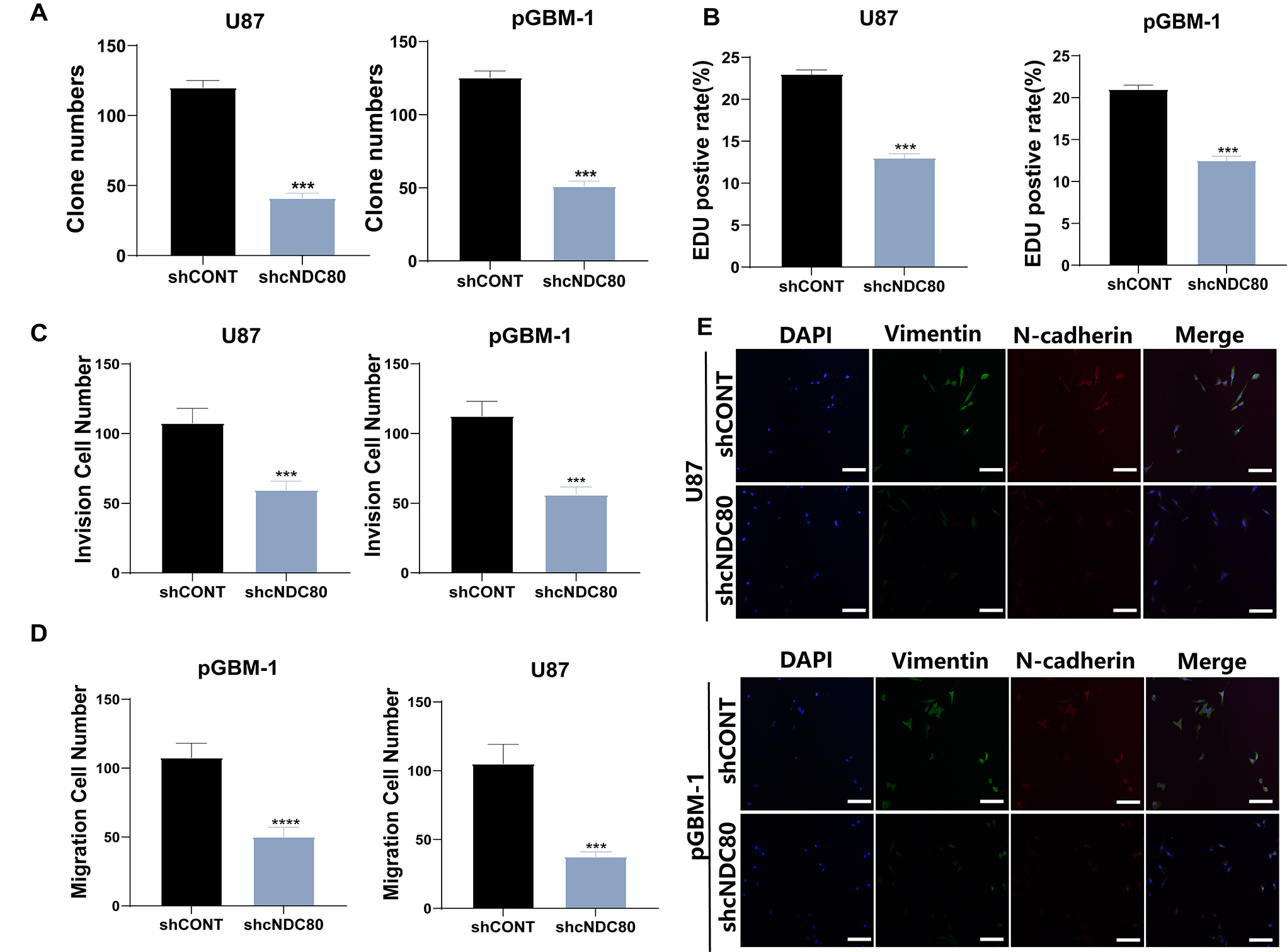

Supplement: Supplementary file 1 — Additional file 1: Figure S1. (A) Statistical bar chart of U87 and pGBM-1 clone formation. (B) Statistical bar chart of EDU positive rate of U87 and pGBM-1. (C-D) Transwell statistical bar chart of U87 and pGBM-1. (E)N-cadherin and Vimentin immunofluorescence staining of GBM cells after treatment. Scale bar, 100 µm. Each experiment was conducted three times, and the findings are shown as mean ± SD. (*P < 0.05, **P < 0.01, ***P < 0.001, ****P < 0.0001). [file 12967_2022_3852_MOESM1_ESM.tif]

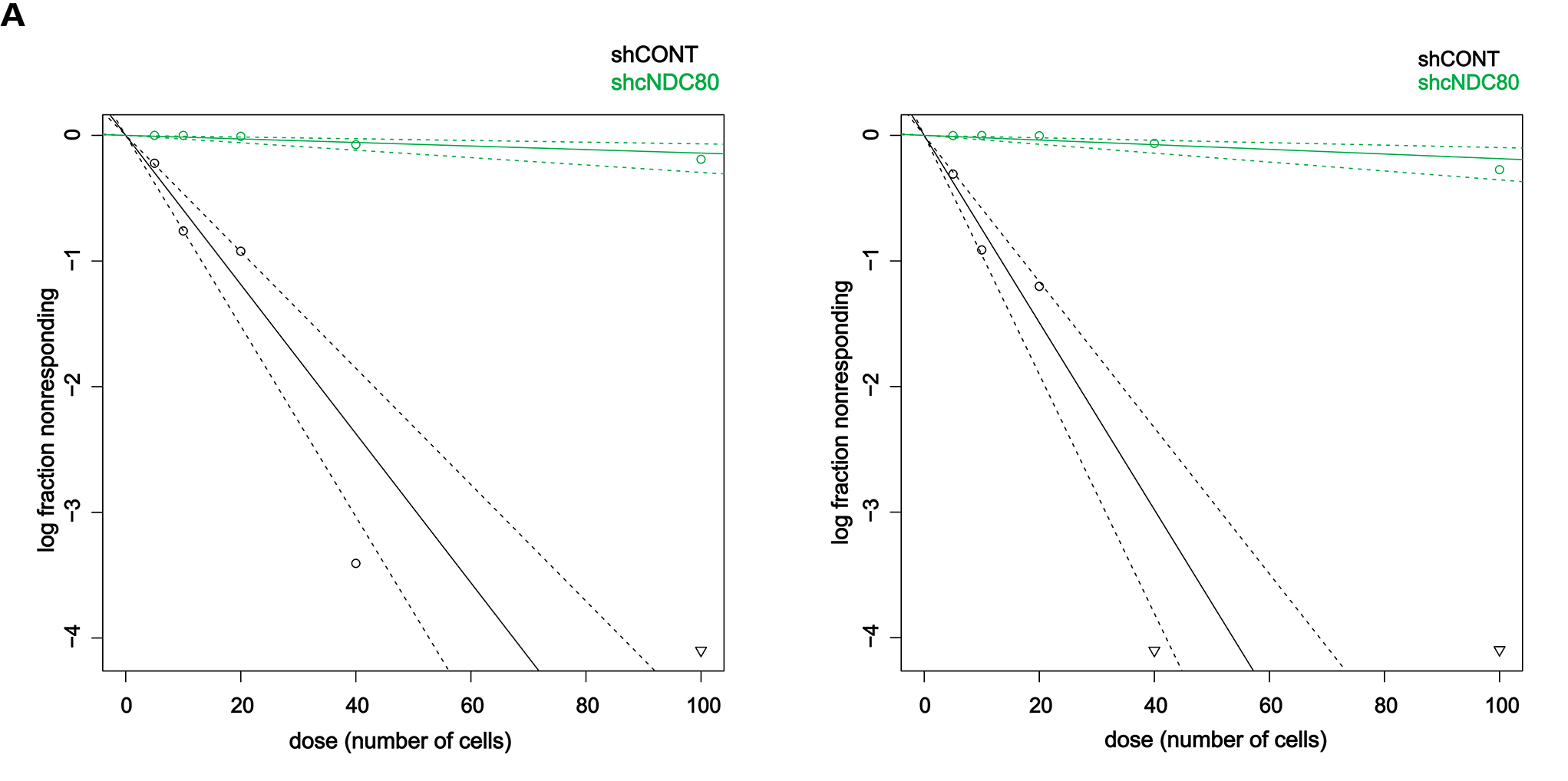

Supplement: Supplementary file 2 — Additional file 2: Figure S2. (A) The capacity of GSCs to proliferate was discovered using the extreme limit dilution test. Each experiment was conducted three times, and the findings are shown as mean ± SD. (*P < 0.05, **P < 0.01, ***P < 0.001, ****P < 0.0001). [file 12967_2022_3852_MOESM2_ESM.tif]

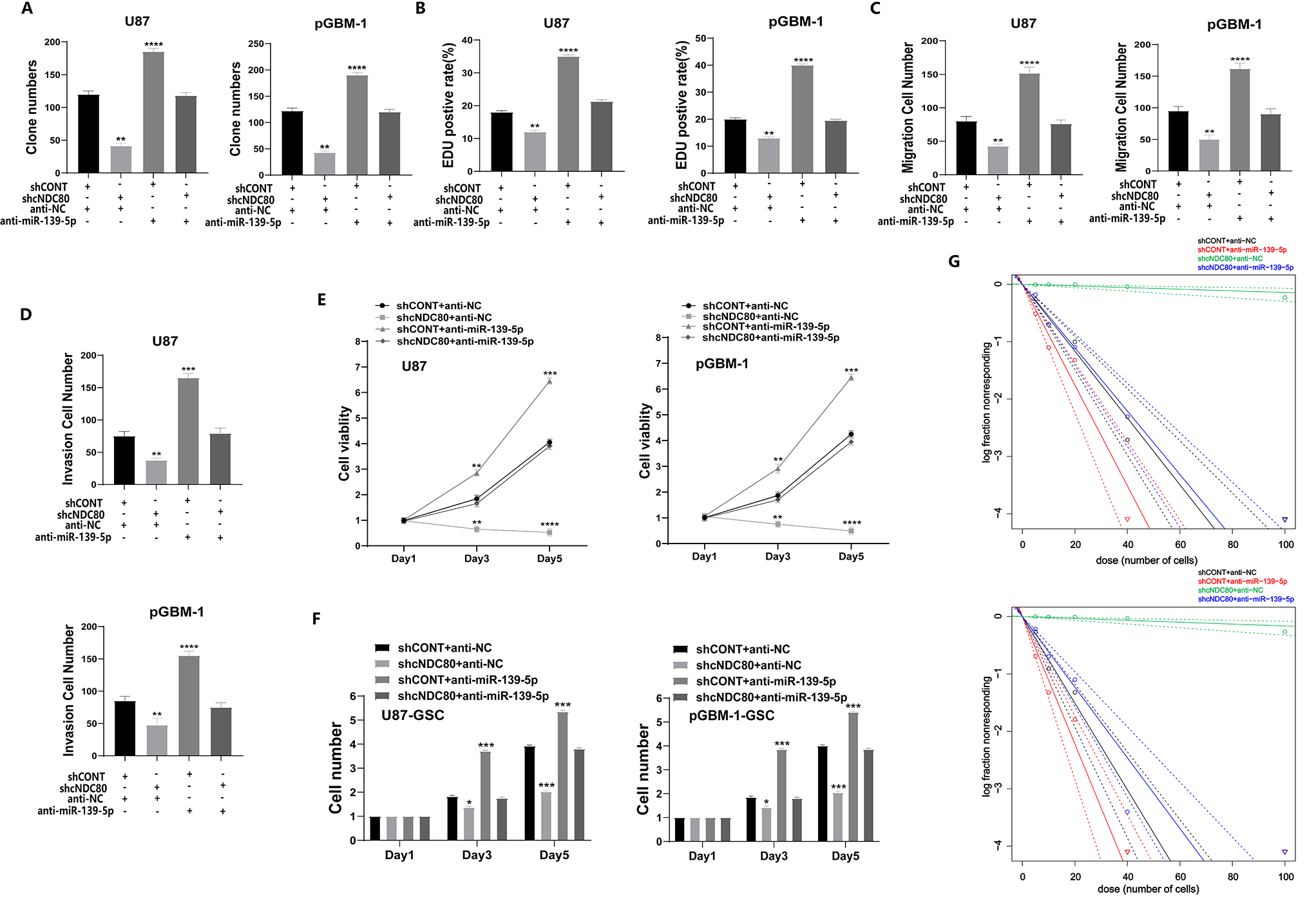

Supplement: Supplementary file 3 — Additional file 3: Figure S3. (A) Statistical bar chart of U87 and pGBM-1 clone formation. (B) Statistical bar chart of EDU positive rate of U87 and pGBM-1. (C-D) Transwell statistical bar chart of U87 and pGBM-1. (E-F) A direct cell count and CellTiter-Glo assay were used to examine the growth and cellular effects of transduced U87-GSCs and pGBM-1-GSCs. (G) The capacity of GSCs to proliferate was discovered using the extreme limit dilution test. Each experiment was conducted three times, and the findings are shown as mean ± SD. (*P < 0.05, **P < 0.01, ***P < 0.001, ****P < 0.0001). [file 12967_2022_3852_MOESM3_ESM.tif]
