# Supplementary material for: CircNDC80 promotes glioblastoma multiforme tumorigenesis via the miR-139-5p/ECE1 pathway
Source: J Transl Med. 2023 Jan 12;21:22. doi: 10.1186/s12967-022-03852-3 (PMC9837923; doi:10.1186/s12967-022-03852-3)
Supplement: Supplementary file 4 — Additional file 4: Table S1. Primers used in the present study. [file 12967_2022_3852_MOESM4_ESM.docx]

**Table S1 Primers used in the present study**

| Gene | Primer sequence |
| --- | --- |
| hsa_circ_0046706 NDC80 | Forward: 5’-TGAAGAATGCATGTCAGAAGA-3’  Reverse: 5’-TGCATTCAATACACTTTACTGAG-3’ |
| NDC80 mRNA | Forward: 5’-CCTCTCCATGCAGGAGTTAAGA-3’  Reverse: 5’-GGTCTCGGGTCCTTGAT TTTCT-3’ |
| MiR-139-5p | Forward: 5’-TCTACAGTGCACGTGTC-3’  Reverse: 5’-CTCAACTGGTGTCGTGG-3’ |
| GAPDH | Forward: 5’-CTCTGCTCCTCCTGTTCGAC-3’  Reverse: 5’-GCGCCCAATACGACCAAATC-3’ |
| U6 | Forward: 5’-GCTTCGGCAGCACATATACTAAAAT-3’  Reverse: 5’-CGCTTCAGAATTTGCGTGTCAT-3’ |
| ECE1 | Forward: 5’-GGACTTCTTCAGCTACGCCTGT-3’  Reverse: 5’-CTAGTTTCGTTCATACACGCACG-3’ |
