# Supplementary material for: CircNDC80 promotes glioblastoma multiforme tumorigenesis via the miR-139-5p/ECE1 pathway
Source: J Transl Med. 2023 Jan 12;21:22. doi: 10.1186/s12967-022-03852-3 (PMC9837923; doi:10.1186/s12967-022-03852-3)
Supplement: Supplementary file 5 — Additional file 5: Table S2. Patient information and diagnostic criteria. [file 12967_2022_3852_MOESM5_ESM.docx]

**Table S2.** Patient information and diagnostic criteria

| **Characteristic** | **Glioma tissues(n=45)** | **Normal brain tissues(n=8)** |
| --- | --- | --- |
| Age, Median (range) | 46, (22-74) | 43, (18-72) |
| Sex |  |  |
| Male | 23 | 4 |
| Female | 22 | 4 |
| Educational level |  |  |
| Low | 10 | 1 |
| Middle | 11 | 3 |
| High | 15 | 3 |
| Other | 9 | 1 |
| Relationship status patient |  |  |
| Not married | 8 | 2 |
| Married or living together | 30 | 5 |
| Divorced | 7 | 1 |
| Tumor type |  |  |
| Low-grade glioma | 15 | - |
| Anaplastic astrocytoma | 18 | - |
| Glioblastoma | 12 | - |
| WHO Grade |  |  |
| II | 15 | - |
| III | 18 | - |
| IV | 12 | - |
| Disease-specific symptoms |  |  |
| Future uncertainty | 33 | - |
| Visual disorder | 15 | - |
| Communication deficits | 29 | - |
| Headaches | 28 | - |
| Seizures | 23 | - |
| Drowsiness | 14 | - |
| Bothered by hair loss | 9 | - |
| Weakness of legs | 7 | - |
| Depressive symptoms | 31 | 3 |
| Fatigue | 23 | 2 |
| Comorbidities |  |  |
| Sinusitis | 14 | 1 |
| Heart disease | 0 | 1 |
| High blood pressure | 4 | 1 |
| Stroke | 2 | 0 |
| Serious bowel issues, longer than 3 months | 6 | 0 |
| Gallstones or gall bladder infection | 7 | 0 |
| Kidney stones | 3 | 0 |
| Diabetes | 6 | 0 |
| Thyroid issues | 8 | 1 |
| Chronic back issues | 2 | 3 |
| Migraine | 4 | 1 |
| Eczema | 1 | 0 |
